# Quick Flicks: Association of Paroxysmal Kinesigenic Dyskinesia and Tics

**DOI:** 10.1002/mdc3.12615

**Published:** 2018-04-17

**Authors:** Bettina Balint, Sarah Wiethoff, Davide Martino, Claudia del Gamba, Anna Latorre, Christos Ganos, Henry Houlden, Kailash P. Bhatia

**Affiliations:** ^1^ Sobell Department of Motor Neuroscience and Movement Disorders, UCL Institute of Neurology Queen Square London UK; ^2^ Department of Neurology University Hospital Heidelberg Germany; ^3^ Neuroimmunology Group, Nuffield Department of Clinical Neurosciences John Radcliffe Hospital Oxford UK; ^4^ Center for Neurology and Hertie Institute for Clinical Brain Research Eberhard Karls‐University Tübingen Germany; ^5^ Department of Molecular Neuroscience, UCL Institute of Neurology Queen Square London UK; ^6^ Department of Clinical Neurosciences University of Calgary Canada; ^7^ Neurology Unit, Department of Clinical and Experimental Medicine University of Pisa; ^8^ Department of Neurology and Psychiatry, Sapienza University of Rome Rome Italy; ^9^ Department of Neurology University Medical Center, Hamburg‐Eppendorf (UKE) Hamburg Germany

**Keywords:** paroxysmal kinesigenic dyskinesia (PKD), tics, PRRT2

## Abstract

**Background:**

Paroxysmal kinesigenic dyskinesia (PKD) is a rare disorder characterised by brief attacks of chorea, dystonia, or mixed forms precipitated by sudden movement.

**Methods:**

Observational study with a cohort of 14 PKD patients and genetic testing for *PRRT2* mutations.

**Results:**

In a series of 14 PKD patients seen in our clinic at the National Hospital of Neurology, Queen Square, from 2012–2017, we noted tics in 11 patients (79%), which stand in stark contrast to the estimated lifetime prevalence of tics estimated to reach 1%.

**Conclusions:**

The two reasons to point out this possible association are the clinical implications and the potential opportunity of a better understanding of shared pathophysiological mechanisms of neuronal hyperexcitability.

## Introduction

Paroxysmal kinesigenic dyskinesia (PKD) is a rare disorder, characterised by brief (seconds to minutes) attacks of chorea, dystonia, or mixed forms precipitated by sudden movement.^1^ Approximately half of the cases are due to autosomal‐dominant *PRRT2* mutations, the phenotypic spectrum of which has broadened to also associate with infantile convulsions with paroxysmal choreoathetosis (ICCA), benign familial infantile epilepsy, and (hemiplegic) migraine and episodic ataxia.^2^ Apart from rare case reports, an association with tics has not been noted so far.^3^, ^4^, ^5^

Tics are rapid, brief, stereotyped movements or vocalizations. They may manifest as eye blinking, shoulder shrugging, grimacing, sniffing, or grunting (simple motor or phonic tics), or as a combined sequence of stereotyped movements or saying words or phrases (complex tics). Typically, tics are (temporarily) suppressible, but patients will describe an inner rising tension or urge. Tics can be associated with different movement disorders, including dystonia.^6^ Here, we describe the association of tics with PKD as observed in a cohort of PKD patients.

## Methods

This was an observational study with a cohort of 14 PKD patients. Genetic testing for *PRRT2* mutations focused on the known mutational hotspot and included Sanger sequencing of all three coding exons and flanking introns of the *PRRT2* gene (*PRRT2*‐001: ENST00000358758).

## Results

In a series of 14 PKD patients (17 to 52 years, median 24 years; 2 females; details in Table 1) seen in our clinic at the National Hospital of Neurology, Queen Square, from 2012 to 2017, we noted tics in 11 patients (79%). All patients were unrelated and had classic PKD with regard age of onset, type, and frequency of attacks and had a good treatment response to antiepileptic drugs. The tics did not differ in their phenomenology from those seen in primary tic disorders, and comprised varying degrees of (mostly simple) motor and vocal tics.7 In general, tics were of mild‐to‐moderate severity and occurred a few to multiple times during the consultations. They were typically not, or only mildly, interfering. All patients could suppress their tics and described an urge while doing so. In all patients, tics were noted prior to treatment with antiepileptic drugs (Table 1). Other features, such as attention deficit hyperactivity disorder and obsessive‐compulsive traits were present in some patients (Table 1). There was no correlation between tics and the *PRRT2* mutational status. Of note, genetic testing for *PRRT2* mutations in this series focused on the known mutational hotspot, which yielded positive results only in a quarter of cases. However, this approach would not exclude other, more rare mutations elsewhere in the gene, the presence of which is not unlikely, given that a classic PKD phenotype (as in our cases) is associated with *PRRT2* gene mutations in up to 90%.^8^

**Table 1:** Characteristics of PKD Patients Seen at Queen Square from 2012–2017

#	Age, Sex	PRRT2 mutation	Epilepsy, migraine	Family history	Tics	Psychiatric comorbidities (OCD, ADHD, other)	Treatment for PKD	Tics present prior to treatment with AED
1	20, M	None detected	Epilepsy age 6m – 3y	‐	+ Simple motor and phonic tics (affecting face, shoulders, hands; sniffing)	‐	CBZ	yes
2	30, M	n.d.	‐	‐	Simple motor tics	OCD	CBZ	yes
3	22, M	c.649dupC p.(Arg217Profs)	migraine	Paternal uncle OCD	Simple motor and phonic tics (facial tics, shoulder elevation, sniffing)	OCD, depression	CBZ	yes
4	27, M	n.d.	‐	‐	Simple motor tics	‐	‐	n.a.
5	28, M	None detected	‐	‐	‐	‐	CBZ, PHT	n.a.
6*	21, M	common C insertion at c.649/p.R217Pfs*8	Epilepsy until age 1.5y	Migraine: mother, maternal grandmother, maternal aunt and brothers; Epilepsy: mother benign infantile febrile seizures	Simple and complex motor tics (excessive blinking, repetitive arm twitching, shoulder shrugging, head tilting/turning, need to touch certain items), simple phonic tics (grunting, sniffing)	OCD, ADHD, Autism spectrum disorder	CBZ	yes
7	19, F	n.d.	‐	‐	Simple motor and vocal tics	‐	CBZ	yes
8	20, M	None detected	Migraine	History of migraine on the paternal side	Simple motor and phonic tics (eye blinking, eye rolling, pulling the side of the mouth; throat clearing)	‐	CBZ	yes
9	32, M	n.d.	‐	Epilepsy and PKD	‐	‐	CBZ	n.a.
10	41, M	None detected	‐	‐	Simple and complex motor tics, simple phonic tics (facial tics, hitting head with hand; a right shoulder jerk; guttural sounds)	‐	‐	n.a.
11	52, M	+ common C insertion at c.649/p.R217Pfs*8	Migraine	Possible family history of epilepsy on maternal side: brother possibly PKD	‐	‐	CBZ	n.a.
12	17, M	n.d.	‐	‐	Simple motor tics and few phonic tics such as sniffs	‐	‐	n.a.
13	24, F	n.d.	Possible infantile seizures	‐	Simple motor tics and phonic tics such as eye blinking, perioral movements, shoulder shrugging, sniffs, throat clearing; complex motor tics such as clenching and unclenching the hands, pulling and tugging at clothes	OCD	CBZ	yes
14	24, F	n.d.	‐	Migraine and tics in her mother	Simple motor tics such as eye blinking, shoulder shrugging; complex motor tics, history of phonic tics like sniffing	OCD	CBZ	yes

^*^previously reported^6^.

Abbreviations: ADHD, attention deficit hyperactivity disorder; CBZ, carbamazepine; n.a., not applicable; n.d., no data on genetic testing/no genetic testing performed; OCD, obsessive‐compulsive disorder, PHT, phenytoin.

## Discussion

The estimated lifetime prevalence of tics has been estimated to reach 1%,^9^ which stands in stark contrast to the observation of tics in 79% of our PKD patients. However, this notion of a possible association is strengthened by previous single case reports,^3^, ^4^, ^5^ describing the occurrence of tics in PKD or PKD‐like patients. The fact that in our cohort, tics were observed in treatment‐naïve patients, argues against the possibility of them being a pharmacological side effect of antiepileptic drug treatment.

The two reasons to point out this possible association of PKD and tics are the clinical implications and the potential opportunity of a better understanding of shared pathophysiological mechanisms.

From a clinical perspective, cooccurring tics might confound the ascertainment of PKD disease burden, more so since both tics and PKD attacks are brief, hyperkinetic movement disorders that may be preceded by sensory symptoms (premonitory urge in tics, aura‐like symptoms in PKD). Given that most of the patients seek medical attention, especially for PKD, the neurologists might overlook the tics. The recognition of cooccurring tics would also lead to different management approaches. Conversely, in patients diagnosed with a tic disorder, the unusual and sudden movements of PKD attacks have been misdiagnosed as complex tics.^4^ Interestingly, the vast majority of our patients was male, and there is a male preponderance in both PKD and tic disorders.^1^, ^7^

To date, one can only speculate about any potential shared mechanisms, which may underlie tics and PKD. Support for the notion of some pathophysiological commonalities between tic disorders and other disorders of neuronal hyperexcitability (like epilepsy) comes from population‐based case‐control studies showing a higher incidence of tics amongst patients with epilepsy,^10^ and an increased risk of epilepsy in children with Tourette's syndrome.^11^ Some authors consider PKD attacks as a form of subcortical epilepsy.^12^ Experimental work showed that intraputaminal or intracortical injections of the GABA‐A antagonist bicuculline cause tics or epilepsy, respectively.^13^, ^14^

*PRRT2* mutations are known to cause epilepsy, and on a neuronal level, to interfere with glutamatergic signalling, thus resulting in neuronal hyperexcitability.^15^ Similarly, recent studies also substantiated evidence of pathological glutamatergic neurotransmission underlying Tourette's syndrome.^16^ Furthermore, there is some evidence suggesting changes in connectivity of certain circuits (cortico‐striato‐pallido‐thalamo‐cortical in tics; thalamomotor‐cortical circuits in PKD with or without *PRRT2* mutations) as shared pathophysiological mechanism.^17^, ^18^

Of course, the small numbers caution to careful consideration of this observation. Larger studies would be needed to conclusively investigate this matter, and considering the relative rareness of PKD, this might require a multi‐centre approach.

## Author Roles

1. Research project: A. Conception, B. Organization, C. Execution; 2. Statistical Analysis: A. Design, B. Execution, C. Review and Critique; 3. Manuscript Preparation: A. Writing of the first draft, B. Review and Critique.

B.B.: 1C, 3A

S.W.: 1C, 3B

D.M.: 3B

C.D.G.: 1C, 3B

A.L.: 1C, 3B

C.G.: 3B

H.H.: 1C, 3B

K.B.: 1A, 1B, 1C, 3B

## Disclosures

**Ethical Compliance Statement**: We hereby confirm that the present study conforms to the ethical standards and guidelines of the journal. The patients have given written and informed consent for online publication of their videos.

**Funding sources and conflicts of interest**: This work was funded by the Medical Research Council (MRC UK), The Wellcome Trust (equipment) and the Synaptopathies strategic award (104033). BB is supported by the EAN research fellowship programme and the Robert Bosch Foundation. The authors don't declare any conflicts of interest concerning the research related to the manuscript.

**Financial disclosures for previous 12 months**: B.B. is supported by the EAN research fellowship programme and the Robert Bosch Foundation. S.W. is supported by the Ministry of Science, Research and the Arts of Baden‐Württemberg and the European Social Fund (ESF) of Baden‐Württemberg (31‐7635 41/67/1). D.M. holds research grants from Immunitaet und Seele Foundation, and has received honoraria/financial support to speak/attend meetings from Allergan. D.M. receives royalties from Springer‐Verlag. C.D.G. and A.L. report no disclosures. C.G. holds research grants from the VolkswagenStiftung (Freigeist Fellowship) and the German Parkinson Society and was also supported by the Deutsche Forschungsgemeinschaft (DFG; GA2031/1‐1 and GA2031/1‐2). H.H. holds research grant from the Medical Research Council (MRC UK), the Wellcome Trust (equipment) and the Synaptopathies strategic award (104033). K.P.B. holds research grants from NIHR RfPB, MRC Wellcome Strategic grant (WT089698) and PD UK (G‐1009), and has received honoraria/financial support to speak/attend meetings from GSK, Boehringer‐Ingelheim, Ipsen, Merz, Sun Pharma, Allergan, Teva Lundbeck and Orion pharmaceutical companies. K.B. receives royalties from Oxford University press and a stipend for MDCP editorship.

## Supporting information

A video accompanying this article is available in the supporting information here.

**Video S1**. Patient 6, who has PKD and carries a mutation of the PRRT2 gene, shows various simple and complex motor tics such as shoulder shrugging, head tilting/turning, perioral movements, drumming the fingers, pulling and tugging at clothes as seen in the video. He can suppress them momentarily with a rising inner tension.

**Video S2**. The first segment shows footage of a PKD attack consisting of a mixture of dystonia and chorea, videoed by the patient herself with a smart phone. The second segment shows the patient in clinic, showing a number of tics, such as eye blinking, grimacing, sniffing, and the ability to supress tics. This video segment starts when she is asked to relax and let any movements occur (0:44). At 0:46 she makes a sniffing sound as a vocal tic, and at 0:46/0:47 she has an eye blinking tic and upper lip elevation. She is then asked to suppress the tics by the videographer (0:55). Between 0:57 and 1:03 she suppresses her tics. She is then asked to relax and let the tics happen at 1:03, and they reappear and you see eye blinking and hear sniffing. She has also these complex tics of wiping her nose with the back of her right hand, and repetitive movement of her hands.
